# Canine corneal endothelial cell analysis using vital dyes and light microscopy

**DOI:** 10.1111/vop.13260

**Published:** 2024-07-16

**Authors:** Yamit Soueid, Shaden Baransy, Yulia Goncharov, Yael Keinan, Lionel Sebbag

**Affiliations:** ^1^ Koret School of Veterinary Medicine The Hebrew University of Jerusalem Rehovot Israel

**Keywords:** alizarin red, corneal edema, corneal endothelial degeneration, corneal endothelial dystrophy, endothelial cell density, trypan blue

## Abstract

**Purpose:**

To evaluate the use of vital dyes and light microscopy for assessing canine corneal endothelial morphology ex vivo.

**Methods:**

The corneas of 40 canine eyes (*n* = 20 dogs) enucleated <24 h following euthanasia or death were isolated and flat‐mounted on a slide. Corneal endothelium was stained via 0.25% trypan blue followed by 0.5% alizarin red (pH 4.2), photographed, then the following morphological features were calculated using ImageJ: mean cell density (MCD), mean cell area (MCA), polymegathism (coefficient of variation of cell area), and pleomorphism (% hexagonality).

**Results:**

Mean ± standard deviation (range) outcomes were: MCD, 2544 ± 541 cells/mm^2^ (1750–3922 cells/mm^2^); MCA, 431 ± 97 μm^2^ (251–626 μm^2^); polymegathism, 17 ± 2% (14%–22%); pleomorphism, 84 ± 3% (80%–90%). No significant differences (*p* ≥ .122) were noted for any outcome between male versus female or brachycephalic versus non‐brachycephalic dogs. Young dogs (<10 years) had lower MCA (*p* = .044), lower pleomorphism (*p* = .003), and higher MCD (*p* = .035) when compared to older dogs (≥10 years). Age was significantly (*p* ≤ .049) correlated with MCA (*r* = 0.467), MCD (*r* = −0.476), polymegathism (*r* = 0.444), and pleomorphism (*r* = 0.609).

**Conclusions:**

The combination of vital dyes and light microscopy allowed for clear visualization and evaluation of the corneal endothelium in canine eyes ex vivo. Our findings can be used in future studies to deepen our understanding of the corneal endothelium in health and disease.

## 1 INTRODUCTION

The corneal endothelium, a neural crest‐derived monolayer of cells located in the posterior aspect of the cornea, is essential for the maintenance of corneal health and transparency by regulating stromal hydration and allowing diffusion of nutrients from the aqueous humor into the cornea.^1^ In dogs, the corneal endothelium can be damaged from primary conditions such as endothelial dystrophy (i.e., the canine equivalent to Fuchs' dystrophy in humans)^2^ and age‐related endothelial degeneration, or secondary conditions affecting the eye such as trauma, uveitis, or glaucoma. Direct observation of the corneal endothelium is not possible; examiners need to rely on diagnostic tools to visualize the corneal endothelium and assess various morphological features such as corneal endothelial cell density, cell area, pleomorphism, and polymegathism.^2^, ^3^, ^4^, ^5^

To date, specular microscopy and in vivo confocal microscopy are the most commonly described tools to assess the corneal endothelium in canine patients.^2^, ^3^, ^4^, ^6^, ^7^ Both imaging modalities provide real time imaging of the corneal endothelium with high resolution images, while confocal microscopy also provides information about other corneal components such as epithelial cells, nerves, and keratocytes.^8^ Both technologies are expensive (albeit specular microscopy is more affordable) and not readily available in most veterinary practices or academic institutions. As such, a cost‐effective technique may help clinicians and scientists when the gold standard imaging techniques are not available, for instance for assessment of corneal endothelial cells ex vivo in donor corneas for transplant (e.g., Descemet's stripping endothelial keartoplasty (DSEK)),^9^ or for evaluation of the corneal endothelial morphology in various species.

In this report, we focus on the combination of vital dyes and light microscopy for ex vivo observation of the corneal endothelium. Following staining of the cornea with vital dyes such as trypan blue and/or alizarin red, the endothelium can be observed using a standard microscope (i.e., light microscopy) for qualitative and quantitative evaluations, as described for human and animal corneas.^10^, ^11^, ^12^, ^13^ To our knowledge, combined staining with trypan blue and alizarin red was only reported in a single canine study to date,^11^ in which the authors focused on a single outcome (i.e., percent hexagonal cells) and did not evaluate other important morphological features described with confocal microscopy or other tools.

## 2 MATERIALS AND METHODS

### 2.1 Animals and samples collection

A total of 40 canine eyes were included in the study, collected from canine patients that died or were euthanized at the authors' institution for reasons unrelated to the study. Signed consent was obtained from all owners. Prior to enucleation, an ophthalmic examination was performed with slit lamp biomicroscopy (SL‐17, Kowa, Japan) by a trained examiner (YS, LS) to exclude eyes with noticeable pathology in the anterior segment such as keratoconjunctivitis, uveitis or cataracts. Both eyes were collected within 24 h of death using a standard subconjunctival approach, then the corneas were processed immediately as described below.

### 2.2 Corneal preparation and staining

The corneas were isolated 360° from the sclera at the level of the limbus using #11 blade and Metzenbaum scissors. Following a gentle rinse with 0.9% NaCl solution to remove any gross debris or blood, four full‐thickness radial incisions were made with Westcott scissors from the peripheral to central cornea (i.e., one incision at each quadrant), and the cornea was mounted on a glass slide with its endothelial side up. The endothelium was then stained with two consecutive vital dyes as previously reported in sheep^12^: (i) trypan blue, prepared as 0.25% solution by mixing the dye (0.4% trypan blue, Sigma Aldrich) with sterile water for injection (Hospira Inc., Lake Forest, IL, USA); and (ii) alizarin red, prepared as 0.5% solution with pH 4.2 by mixing the dye (Alizarin Red S, Sigma Aldrich) with sterile water for injection, then reaching the desired pH by adding minute amounts of hydrochloride acid. Notably, both solutions were prepared fresh on a monthly basis by a pharmacist and were kept refrigerated at 4°C between uses. If dyes were used >4 weeks following preparation, staining quality was subjectively reduced which could have affected proper morphological evaluation of the corneal endothelium (data not shown). The dual staining was performed as follows: First, the endothelium was covered with 0.25% trypan blue solution for 90 s; second, following a gentle rinse of the cornea with saline, the endothelium was covered with 0.5% alizarin red solution for 90 s and again gently rinsed with saline.

### 2.3 Image capturing and data collection

The stained corneal endothelium was observed at 20× magnification using light microscopy. Three photographs were taken from the central aspect of each cornea with an image capture system, then all images were evaluated for the following morphological features using ImageJ program:

- Mean cell density (MCD) in cell/mm^2^—All cells were manually counted within a region of interest (ROI) delineated by a 20 000 μm^2^ rectangular area in each image. Of note, cells overlapping the ROI borders were counted only on one pair of adjacent borders (e.g., upper and left, lower and right) and discarded on the other pair, as previously described.^3^ The MCD was then calculated as the total number of cells divided by the ROI size.
- Mean cell area (MCA) in μm^2^—Fifty cells from each photograph were chosen randomly in each image. The borders of each cell were manually marked, then cell area (μm^2^) was measured by clicking the “measure” button in the ImageJ program. Mean (MCA) and standard deviation of the 50 cells areas were automatically calculated by the software.
- Polymegathism in %—Polymegathism is defined as the coefficient of variation of cell area. The percentage of cells displaying polymegathism was calculated by dividing the standard deviation of cell area with the MCA.
- Pleomorphism in %—Pleomorphism is the percentage of endothelial cells with a regular hexagonal pattern. Pleomorphism was calculated as the ratio of cells with 6 sides (manually counted) out of 50 randomly selected cells in each image.

### 2.4 Data analysis

Normality of data was evaluated using the Shapiro–Wilk test. Paired *t*‐tests were used to compare results between the right and left eyes; since no statistical differences were observed for any outcome (*p* ≥ .186), average values of both eyes were used for further analysis. Student's *t*‐tests were used to compare results of each outcome between male versus female dogs, brachycephalic versus non‐brachycephalic dogs, and young (<10 years old) versus old (≥10 years old) dogs. Last, potential associations between age and endothelial morphological features were assessed with Pearson's correlation tests; results of Pearson's tests were interpreted following guidelines described by Campbell and Swinscow^14^: very weak (0–0.19), weak (0.2–0.39), moderate (0.40–0.59), strong (0.6–0.79), and very strong (0.8–1.0). Statistical analysis was performed with SigmaPlot version 15.0 (Systat Software Inc., San Jose, CA), with *p* values lower than .05 considered significant.

## 3 RESULTS

**FIGURE 1:**
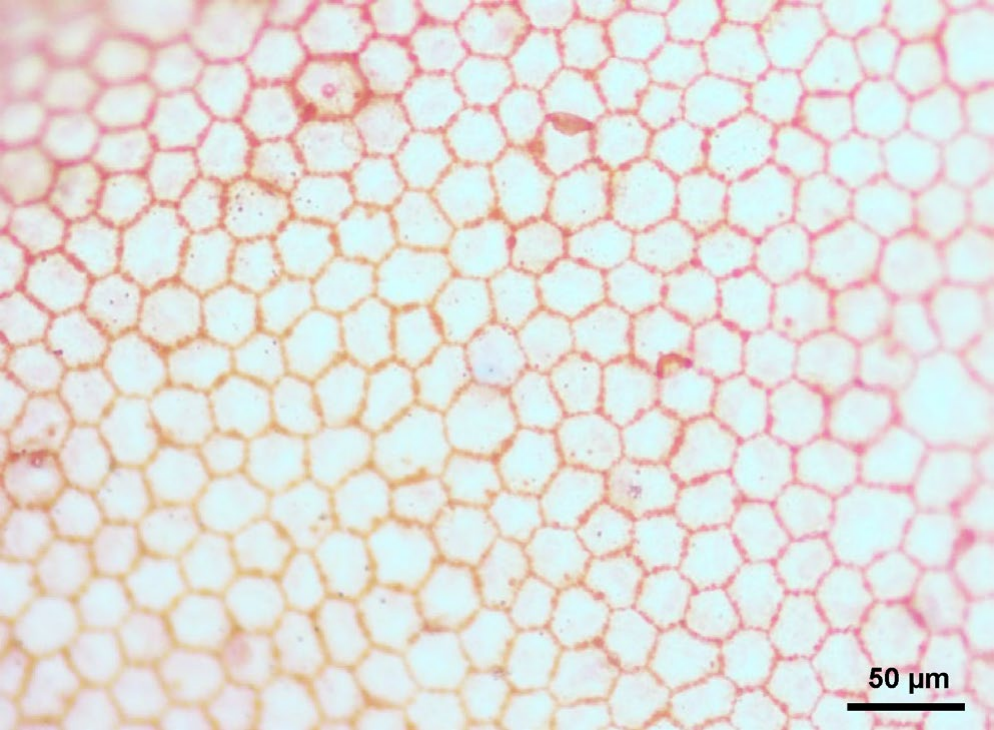
Representative photomicrograph of the canine corneal endothelium observed with light microscopy (20× magnification) following staining with 0.25% trypan blue and 0.5% alizarin red dyes.

**FIGURE 2:**
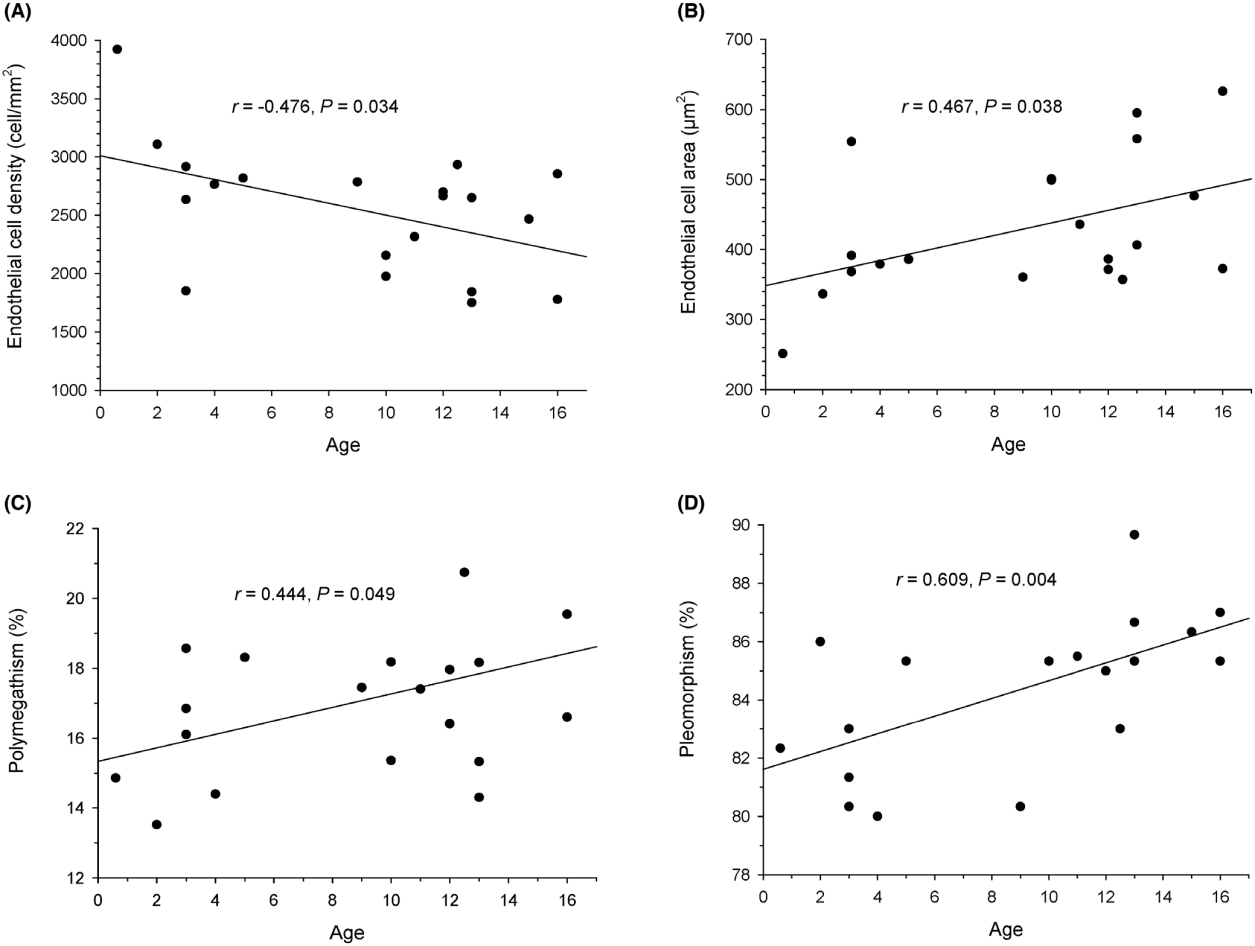
Results of the Pearson correlation testing between the dogs age and the following corneal endothelium parameters: endothelial cell density (A), endothelial cell area (B), polymegathism (C), and pleomorphism (D).

Data were normally distributed (*p* ≥ .154); therefore, results are presented as mean ± standard deviation (range). Table 1 describes the study population. A representative image of the stained corneal endothelium in a cadaver canine eye is shown in Figure 1, while the overall study results are summarized in Table 2. No statistical differences were observed for any outcome between male versus female dogs (*p* ≥ .248) or brachycephalic versus non‐brachycephalic dogs (*p* ≥ .122). Young dogs had significantly lower endothelial cell area (*p* = .044), lower pleomorphism (*p* = .003), and higher endothelial cell density (*p* = .035) when compared to old dogs. Polymegathism was also lower in young versus old dogs, but the difference was not statistically significant (*p* = .148). Significant correlations were observed between age and endothelial morphological features (Figure 2), namely, moderate positive association between age and endothelial cell area (*r* = 0.467, *p* = .038), moderate negative association between age and endothelial cell density (*r* = −0.476, *p* = .034), moderate positive association between age and polymegathism (*r* = 0.444, *p* = .049), and strong positive association between age and pleomorphism (*r* = 0.609, *p* = .004). Two examples of damaged corneal endothelium from diseased canine eyes are described in Figures 1 and 2 of the Appendix S1.

**TABLE 1:** Demographics and cause of death or euthanasia of the study population.

	Breed	Sex	Age (years)	Weight (kg)	Cause of death or euthanasia
1	Shih Tzu	MN	4	7	Hit by car
2	Mixed	FS	11	32	Abdominal neoplasia, acute kidney injury
3	Miniature Schnauzer	MI	13	5	Splenic hemangiosarcoma
4	Golden Retriever	FS	16	25	Dyspnea, epistaxis, severe anemia
5	Toy Poodle	FS	5	4	Hit by car
6	Cocker Spaniel	MN	9	8	Congestive heart failure
7	Mixed	FS	9	–	Splenic hemangiosarcoma
8	Belgian Shepherd	MI	3	22	Anemia, hypovolemic shock
9	Mixed	FS	13	–	Gastric mass, hypovolemic shock
10	Corgi	MI	2	10	Hit by car
11	Labrador Retriever	MN	15	30	Progressive weakness
12	Shih Tzu	FI	3	6	Hepatic mass, pancreatitis
13	Jack Russell Terrier	MN	12	4	Hemoabdomen
14	Pointer	FS	10	28	Diffuse mast cell tumor
15	Mixed	MN	13	17	Lymphoma
16	Shih Tzu	FS	12	4.5	Chronic kidney disease
17	Mixed	MN	12	25	Hemoabdomen
18	Mixed	MI	0.5	7	Vertebral fracture
19	Siberian Husky	FS	16	23	Liver mass, hemoabdomen
20	Cavalier King Charles Spaniel	FS	10	9	Congestive heart failure

Abbreviations: FI, female intact; FS, female spayed; MI, male intact; MN, male neutered.

**TABLE 2:** Mean ± standard deviation of various corneal endothelium parameters in 40 cadaver canine eyes, depicting with an asterisk (*) any significant differences *p* < .05 between the following groups: Male versus female, brachycephalic versus non‐brachycephalic, young (< 10 years old) versus old (≥10 years old) dogs.

	Endothelial cell area (μm^2^)	Endothelial cell density (cells/mm^2^)	Polymegathism (%)	Pleomorphism (%)
All	431 ± 97 (251–626)	2544 ± 541 (1750–3922)	17 ± 2 (14–22)	84 ± 3 (80–90)
Male	408 ± 105 (251–595)	2687 ± 616 (1750–3922)	17 ± 3 (14–22)	84 ± 3 (80–90)
Female	454 ± 88 (368–626)	2401 ± 440 (1777–2917)	17 ± 1 (15–20)	85 ± 2 (80–87)
Brachycephalic	436 ± 90 (360–595)	2451 ± 454 (1750–2917)	16 ± 2 (14–19)	84 ± 3 (80–90)
Non‐brachycephalic	444 ± 95 (336–626)	2491 ± 461 (1777–3108)	18 ± 2 (14–22)	85 ± 2 (81–87)
Young (<10 years old)	378 ± 84 (251–554)	2850 ± 570 (1852–3922)	16 ± 2 (14–19)	82 ± 2 (80–86)
Old (≥10 years old)	465 ± 92* (357–626)	2299 ± 391* (1750–2855)	18 ± 2* (14–22)	86 ± 2* (83–90)

## 4 DISCUSSION

The present study assessed the canine corneal endothelium ex vivo by using vital dyes and light microscopy, describing a technique that can be used in future studies to deepen our understanding of the corneal endothelium in health and disease. Alizarin red dyes the edges of endothelial cells and the areas of cell loss where Descemet's membrane is exposed, while trypan blue can help identify degenerated and dead corneal endothelial cells by penetrating damaged cell membranes and dying the cell nuclei blue.^10^, ^15^ In the present study, it was possible to clearly identify the endothelial cell borders stained in red by the alizarin red dye, providing quality images that were used to evaluate morphological features of the canine endothelial cells. Alizarin red also highlighted a few areas denuded of endothelium by staining the underlying Descemet membrane; this finding was observed sporadically in the corneal endothelium of pathology‐free canine eyes, and more commonly in the few diseased eyes that we examined (see Appendix S1), albeit objective evaluation of these denuded lesions was beyond the scope of the present study. The use of trypan blue allowed for the identification of damaged endothelial cells in the few diseased eyes that were examined, often neighboring areas of denuded endothelium that were highlighted with alizarin red. When noted, the blue staining of the endothelial cell nuclei varied in intensity; this finding may be important to consider in future studies, as one report classified endothelial cells that had “very faint” blue staining “nonpositive,” presuming the cell damage might be too small to affect the cell viability.^15^

The technique reported in the present study could be used in various clinical or research settings, especially when the gold standard tools are not available. As reported once in dogs,^9^ vital staining could be used ex vivo to assess the suitability (or lack thereof) of corneal donors prior to DSEK surgery in canine patients. However, trypan blue alone (and not alizarin red) was used in that canine study; therefore, it is critical for additional studies to investigate in vivo viability of alizarin red before using dual staining in clinical practice. Further, the technique could be used to assess the corneal endothelium following enucleation of canine patients with various ocular pathologies (e.g., uveitis, glaucoma), as exemplified in two cases from the present study (see Appendix S1). Last, the technique could be used to evaluate the corneal endothelial morphology in various species postmortem, for instance in wildlife or exotic animals in whom data collection in vivo is often limited by challenges in animal's restraint and safety. For all the aforementioned uses, the authors believe the ex vivo data could provide a reasonable estimate of the “true” corneal endothelial morphology if it was assessed in vivo, although further experiments are required to compare the two methods in the same eyes. In fact, the study's findings compared well with in vivo methods previously described in dogs for most outcomes evaluated, indicating a minimal/insignificant impact of postmortem changes (e.g., tissue shrinkage, postmortem cellular alterations) on corneal endothelial morphology when assessing freshly enucleated eyes. Corneal endothelial cell density averaged 2544 cells/mm^2^ in our canine population, a finding that was quite similar with reported values from healthy dogs when using specular microscopy (2176–2635 cells/mm^2^)^7^, ^16^, ^17^, ^18^ and confocal microscopy (2118–3175 cells/mm^2^).^2^, ^3^, ^4^ Corneal endothelial cell area averaged 431 μm^2^ in the present study, comparable to the report by Datiles et al that used specular microscopy (385 μm^2^).^17^ Polymegathism averaged 17% in our canine population, similar to Miyagi et al. findings when using manual assessment of confocal microscopy images (17.3%).^3^ In regard to pleomorphism, the average value in our study (84%) was larger than reported values with confocal microscopy (61.4%)^3^ and specular microscopy (57%–73%)^17^, ^18^; in other words, more endothelial cells appeared hexagonal when assessed ex vivo with light microscopy than in vivo with confocal or specular microscopy. The reason for this discrepancy is unclear but may be related to tissue distortion when the endothelium is assessed ex vivo, or simply due to studies differences in the canine population studied (e.g., breed, age) or the methodology in quantifying pleomorphism (e.g., corneal location, number of cells examined).

Age significantly impacted the corneal endothelial morphology in the present canine study, similar to findings from other reports in dogs,^4^, ^7^, ^19^ humans,^20^ and other species.^21^, ^22^, ^23^ In particular, the density of corneal endothelial cells decreased with advancing age—likely due to age‐related cell loss and limited proliferative capacity of the corneal endothelium^24^—a finding that was observed with light microscopy (present work), confocal microscopy,^4^ specular microscopy,^7^ and scanning electron microscopy.^19^ With advancing age and reduced cell density, the area of remaining cells increases to cover the same surface with fewer endothelial cells, a phenomenon that was confirmed in the present experiment. Further, pleomorphism and polymegathism increased with advancing age in our canine population, as showed in a previous study using ex vivo scanning electron microscopy to assess the corneal endothelial in dogs.^19^ Variables other than age do not appear to influence the corneal endothelial morphology in dogs, including laterality (right vs. left eye),^7^ sex (male vs. female), or cephalic conformation (brachycephalic vs. non‐brachycephalic).

Ex vivo evaluation of the corneal endothelium presents several advantages. First, the technique reported herein is relatively inexpensive and could therefore become available to clinicians and scientists who do not have access to expensive equipment such as confocal microscopy or specular microscopy. Second, since the endothelium is observed directly (‘en face’) and not through the corneal epithelium and stroma, the technique is presumably not affected by corneal edema or other pathologies that could mask the view of the corneal endothelium when imaging the cornea from outer to inner surfaces^2^, ^16^; in fact, endothelial cell density could not be assessed by confocal microscopy in several dogs diagnosed with corneal endothelial dystrophy due to severe corneal edema.^2^ Third, vital staining and light microscopy of the corneal endothelium might be able to detect cytological and morphological details that would be otherwise be missed with corneal imaging; for instance, excrescences of extracellular matrix on the Descemet membrane (i.e., corneal guttae, a hallmark of Fuchs' dystrophy in humans) could not been observed with in vivo confocal microscopy of dogs with corneal endothelial dystrophy,^2^ but could potentially be detected with vital staining that would bind and highlight the extracellular matrix. Such speculations should be verified in future studies comparing different methods in healthy and diseased canine eyes.

On the other hand, ex vivo assessment of the corneal endothelium presents several disadvantages. First, corneal endothelial cells degenerate postmortem, a process that could affect the reliability of selected measurements; as such, care should be taken to collect and process the corneal samples shortly after death or euthanasia, as conducted in the present study (<24 h) and other ex vivo reports.^11^, ^12^, ^25^ Second, tissue distortion and/or shrinkage could occur from processing and fixation of the corneal specimens. In fact, when canine corneal specimens were extensively processed for scanning electron microscopy (e.g., fixation in glutaraldehyde, post‐fixation in osmium tetroxide, dehydration in ethanol, etc.), quantified endothelial cell densities were much higher (3700–17 000 cells/mm^2^) than reported values in other canine reports.^19^ Tissue shrinkage attributable to processing may decrease individual cell size and thereby increase endothelial cell density.^26^ Third, ex vivo assessment cannot provide information for longitudinal studies, unlike in vivo methods such as confocal microscopy.^2^ Last, corneal sampling and processing may be tedious and more time‐consuming with ex vivo techniques, especially when compared to corneal imaging performed in vivo by experienced investigators in awake animals. It is possible that newer tools will assist investigators to expedite and optimize analysis of collected data, for instance deep learning‐based application for automated morphometric analysis of corneal endothelial cells,^27^ rendering ex vivo assessment more widely applied in practice.

The main limitation of the study is the focus on a single protocol for dying the corneal endothelium in our canine subjects. Here, the trypan blue concentration (0.25%) was based on two previous reports^10^, ^12^ and was purposely higher than a previous canine report by Boo et al (0.06%) where no noticeable blue staining could be observed in the reported endothelial images.^9^ For the alizarin red concentration (0.5%), the endothelial images obtained by Park et al. with 0.5% seemed sharper than the 1% concentration from the same report,^15^ as well as the lower concentration used in previous reports (0.2%).^11^, ^12^ As mentioned in the methods section, the pH of the alizarin red solution was adjusted to 4.2 with hydrochloride acid in order to optimize dye staining at the intercellular borders.^28^ Although hydrochloride acid can be toxic to the corneal endothelium, only a small amount was added to the large volume of alizarin red solution, and the contact time with the cornea was short (90 s); therefore, we believe the potential impact on endothelial morphology would be negligible, if any. In fact, we did not observe any gross toxic changes to the corneal endothelium in our corneal specimens. Nonetheless, the dying method was empirically selected and may be suboptimal for the evaluation of the corneal endothelium in dogs. In fact, previous literature reported dye concentrations varying from 0.2%–1% for alizarin red and 0.001%–0.5% for trypan blue, various exposure times, and different staining methods such as dropwise instillation (as done in our study) or tissue immersion in the dye.^15^ Another methodology limitation is the use of sterile saline for compounding the dyes and rinsing the corneal endothelium during the dying process, a solution that could have been irritating to the corneal endothelium and affect some of the morphological results; future experiments should consider balanced salt solution as reported in other studies.^9^, ^12^ Last, the study relied on corneal flat mounting for assessing the endothelium, as previously described^11^, ^12^; although there were sufficient areas that could be imaged for data analysis, the excess tissue wrinkling limited the view of large segments in each eye, a pitfall that could potentially be minimized by harvesting a corneal button (e.g., 7‐mm trephine)^29^ to optimize the field for light microscopy.

In summary, the association of alizarin red, trypan blue, and light microscopy allowed for clear visualization of the corneal endothelium in dogs, providing insight into the morphology of corneal endothelial cells in health and disease. Future studies could optimize the vital staining methodology for canine eyes, compare vital dyes to other tools, optimize data analysis (e.g., manual vs. automated assessment, deep learning tools),^3^, ^13^, ^27^ assess other animals, and evaluate the impact of various ocular pathologies on the corneal endothelium.

## AUTHOR CONTRIBUTIONS

**Yamit Soueid:** Conceptualization; investigation; writing – review and editing. **Shaden Baransy:** Formal analysis; investigation. **Yulia Goncharov:** Investigation. **Yael Keinan:** Formal analysis. **Lionel Sebbag:** Conceptualization; formal analysis; investigation; supervision; writing – original draft; writing – review and editing.

## CONFLICT OF INTEREST STATEMENT

The authors declare no conflict of interest.

## ETHICS STATEMENT

Informed consent was obtained from the animals' owners, and the study adhered to the Guidelines for Ethical Research in Veterinary Ophthalmology (GERVO).

## Supporting information


Appendix S1.

## Data Availability

The data that support the findings of this study are available from the corresponding author upon reasonable request.
