# Supplementary material for: Canine corneal endothelial cell analysis using vital dyes and light microscopy
Source: Vet Ophthalmol. 2024 Jul 16;28(4):675–82. doi: 10.1111/vop.13260 (PMC12274124; doi:10.1111/vop.13260)

APPENDIX

Figure 1. Photomicrograph of the corneal endothelium in a 3-year-old Shih Tzu following traumatic proptosis and enucleation, as observed with light microscopy (20X magnification) following staining with 0.25% trypan blue and 0.5% alizarin red dyes. Notice the blue coloration of the nuclei, indicating endothelial cells damage, and the red coloration of the Descemet membrane in areas denuded of endothelial cells (arrowheads).

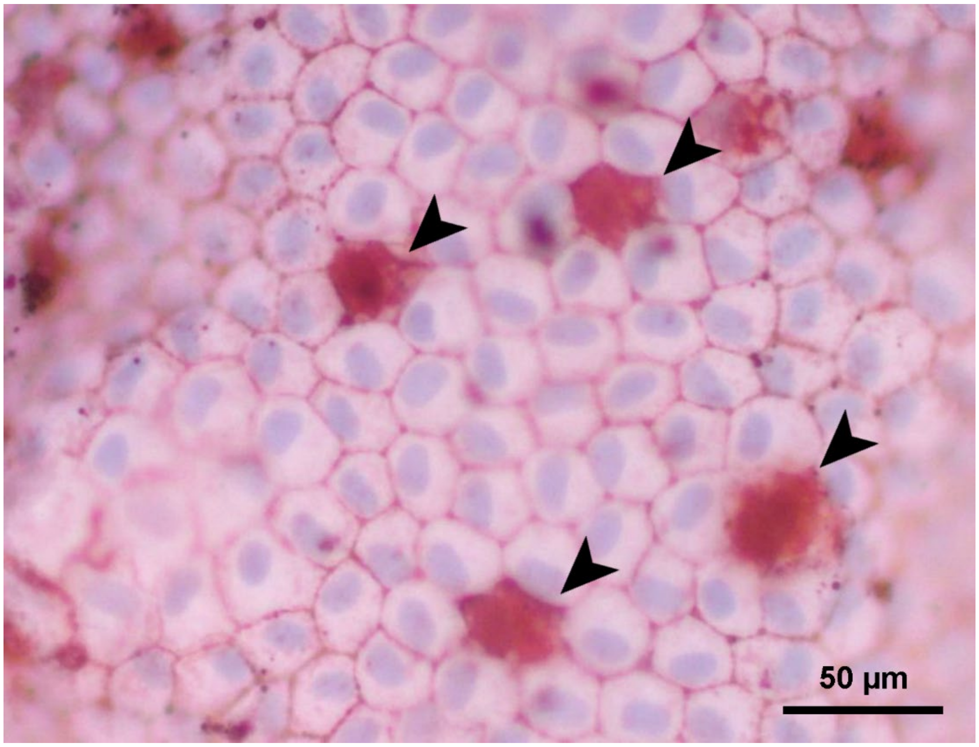

Figure 2. Photomicrograph of the corneal endothelium in an 11-year-old male castrated Pekingese following enucleation due to glaucoma, as observed with light microscopy (20X magnification) following staining with 0.25% trypan blue and 0.5% alizarin red dyes. Notice the blue coloration of the nuclei, indicating endothelial cells damage, and the red coloration of the Descemet membrane in areas denuded of endothelial cells (arrowheads).

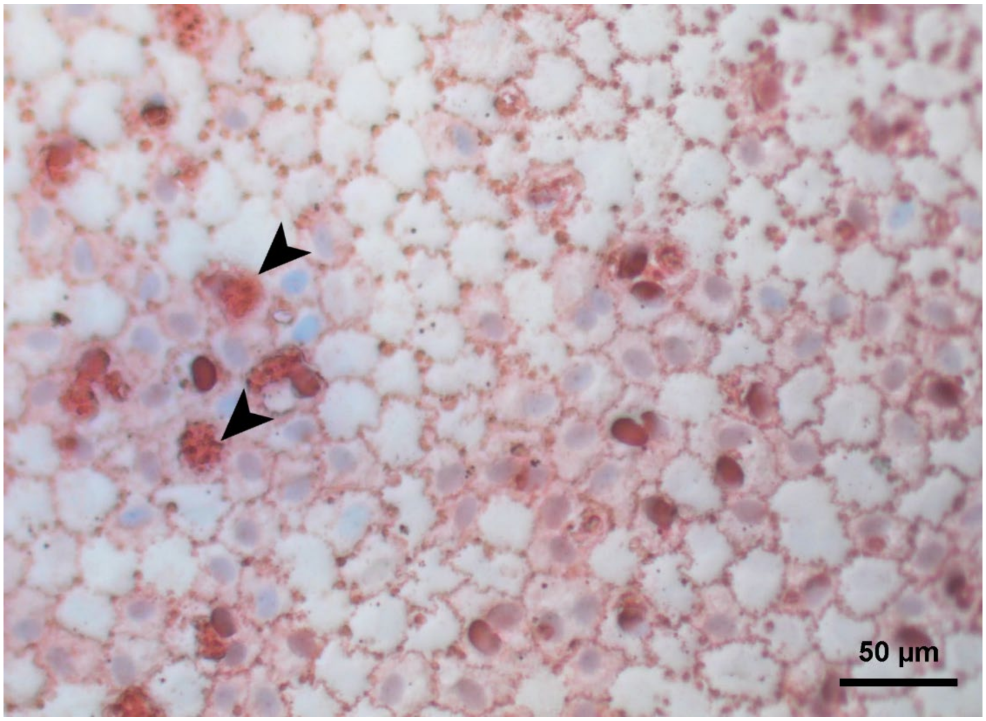

Supplement: Supplementary file 1 — Appendix S1. [file VOP-28-675-s001.pdf]
